# Effectiveness of an mHealth Exercise Program on Fall Incidence, Fall Risk, and Fear of Falling in Nursing Home Residents: The Cluster Randomized Controlled BeSt Age Trial

**DOI:** 10.3390/sports14010041

**Published:** 2026-01-15

**Authors:** Jonathan Diener, Jelena Krafft, Sabine Rayling, Janina Krell-Roesch, Hagen Wäsche, Anna Lena Flagmeier, Alexander Woll, Kathrin Wunsch

**Affiliations:** 1 Institute of Sports and Sports Science, Karlsruhe Institute of Technology, 76131 Karlsruhe, Germany; 2 Department of Sport Science, University of Koblenz, 56070 Koblenz, Germany; 3 AOK Baden-Württemberg, 70191 Stuttgart, Germany

**Keywords:** nursing home residents, mHealth, fall prevention, physical activity, balance, mobility, older adults, randomized controlled trial

## Abstract

The global rise in nursing home (NH) populations presents substantial challenges, as residents frequently experience physical and cognitive decline, low physical activity, and high fall risk. This study evaluates the effectiveness of the BeSt Age App, a tablet-based, staff-supported mHealth intervention designed to promote physical activity and prevent falls among NH residents. Primary outcomes were fall incidence and fall risk (assessed using Berg Balance Scale [BBS] and Timed Up and Go [TUG]); fear of falling was a secondary outcome. In a cluster-randomized controlled trial across 19 German NHs, 229 residents (mean age = 85.4 ± 7.4 years; 74.7% female) were assigned to an intervention group (IG) or control group (CG). The 12-week intervention comprised twice-weekly, tablet-guided exercise sessions implemented by NH staff. Mixed models and generalized estimating equations were used under an intention-to-treat framework. The IG showed significantly greater improvement in BBS scores than the CG (group × time: *F*(1, 190.81) = 8.25, *p* = 0.005, *d* = 0.22), while group × time changes in TUG performance, fear of falling, and fall incidence were nonsignificant. These findings demonstrate the feasibility of a staff-mediated mHealth approach to fall prevention in NH residents, showing significant improvements in BBS scores as one functional indicator of fall risk, while TUG, fall incidence and fear of falling showed no change.

## 1. Introduction

The aging of populations worldwide and the rising number of individuals residing in nursing homes (NHs) pose major challenges for health care systems. NH residents frequently experience age-related physical and cognitive decline, mobility impairments, and chronic conditions, all of which contribute to reduced physical activity (PA) and an elevated risk of falls [1,2]. Compared to community-dwelling older adults, NH residents are less active [3,4] and experience about twice the fall rate of age-matched cohorts in the community [5]. A Canadian study reported that 62% of NH residents experience at least one fall annually [6]. In Germany, 5.5% of residents experience a fall within just two weeks [7], and annual rates exceed 1.5 falls per resident [8].

One-quarter of falls in NHs result in hospitalization [9]. In addition to injuries, falls often lead to increased fear of falling, reduced confidence in daily activities, and withdrawal from PA [10]. This cycle may contribute to frailty, social isolation, depression, and diminished quality of life [11,12].

Engagement in regular PA is one of the most effective strategies to mitigate these detrimental consequences. The World Health Organization recommends that older adults perform 150–300 min of moderate-to-vigorous aerobic activity weekly, combined with strength training at least twice a week and balance-focused activities three times a week to reduce fall risk [13]. In addition, expert taskforces have developed specific guidelines for long-term care facilities, emphasizing the need to reduce sedentary behavior and recommending multicomponent exercise programs (strength, endurance, balance, and flexibility) at moderate intensity, twice weekly, in structured sessions [14].

Exercise interventions meeting these recommendations have been shown to improve mobility, balance, and strength, thereby reducing fall risk in NH residents [15,16]. They also provide wider health benefits, including reductions in depression, anxiety, and fear of falling, and improvements in quality of life [17,18]. However, overall evidence regarding the effectiveness of exercise interventions in NH settings remains inconclusive. Recent trials and reviews indicate that multicomponent or balance-focused programs reduce falls or improve physical performance, whereas others show no effect [19,20,21]. This inconsistency has been linked to heterogeneity in intervention content, exercise dose and duration, differences in study design and outcome measures, and difficulties achieving sufficient adherence particularly in very frail or cognitively impaired residents [19,20,21,22]. Another reason may be the considerable heterogeneity among residents in physical and cognitive functioning [23,24,25].

Addressing this variability among NH residents requires intervention approaches that are both adaptable and inclusive. In this context, digital health technologies offer promising opportunities to deliver individualized and scalable exercise programs in NH settings. Electronic and mobile health (mHealth) applications allow programs to be adapted to residents’ individual needs and can help overcome barriers such as low motivation or limited knowledge about exercise [26]. Despite this potential, most digital approaches implemented in NHs to date have focused on exergaming [27]. While exergaming has demonstrated positive effects on balance and fall reduction in some older adult populations [28,29,30], its feasibility in NH residents is limited. Barriers to using exergaming technologies include unfamiliarity with equipment, complex controls, and unclear instructions, which can be challenging for older adults [31]. This is particularly problematic for residents with advanced physical or cognitive impairments, meaning that those at highest risk of falling are often excluded [32]. Such barriers thus highlight the need for approaches that are staff supported rather than complex user operated systems, enabling wider participation among NH residents.

To address these challenges, we developed the BeSt Age App, a tablet-based intervention that can be used by NH staff to deliver individualized, multicomponent exercise and fall prevention programs specifically designed for NH residents. The app supports staff in forming groups of residents with similar levels of cognitive and motor abilities, tailoring and delivering exercise sessions to participants’ needs, thereby promoting broader participation. This cluster-randomized controlled trial (cRCT) aimed to evaluate the effectiveness of the intervention in reducing fall incidence, lowering fall risk, and decreasing fear of falling among NH residents. We hypothesized that the intervention would lead to a reduction in fall incidence, improved balance and mobility as indicators of fall risk, and reduced fear of falling compared with the control group.

## 2. Materials and Methods

This study was preregistered in the German National Register of Clinical Trials (DRKS00032349). Ethical approval was obtained from the ethics committee of the Karlsruhe Institute of Technology, Karlsruhe, Germany. A cRCT was conducted in NHs in Southern Germany. NHs were randomly assigned to either the intervention group (IG) or the control group (CG). Randomization was conducted at the facility level because the group based matching algorithm implemented in the app requires a sufficient number of residents within each facility to form homogeneous exercise groups, which would not have been feasible under individual randomization. No stratification or minimization procedures were used in the cluster randomization. Further information about the rationale and details on the development process of the intervention can be found in the study protocol for the BeSt Age intervention [33], which also outlines the methodological background. The present manuscript provides detailed statistical analysis and reports the effectiveness of the cRCT with regard to fall incidence, fall risk and fear of falling.

### 2.1. Participants

NHs in the city of Karlsruhe and the surrounding districts in the state of Baden-Württemberg, Germany, were recruited through telephone outreach and on-site informational meetings. Inclusion criteria for NH residents were as follows: (1) Age above 65 years, (2) at least 50% functional capacity of the extremities (i.e., at least two of four limbs: both arms, both legs, or one arm and one leg), and (3) ability to follow instructions. The 50% functional capacity criterion ensured that residents could safely perform the movements required for the exercise program, receive a sufficient training stimulus to allow for potential functional improvement, and complete most of the functional outcome assessments. Functional capacity was evaluated by the research team using a brief standardized screening of upper and lower limb mobility to determine whether participants met the ≥50 percent capacity threshold. Upper limb capacity was defined as the ability to actively lift each arm to approximately 90 degrees of shoulder flexion (i.e., shoulder height), and lower limb capacity as the ability to perform a seated knee extension to approximately 90 degrees. Exclusion criteria were very severe cognitive, neurological, or motor impairments. Eligibility of participants was verified at baseline assessment based on these inclusion and exclusion criteria. Written informed consent was obtained from all participants or their legal guardians prior to the study.

### 2.2. Intervention

The BeSt Age intervention consisted of a tablet-based exercise program implemented by NH staff to promote PA among residents with varying motor and cognitive abilities. We developed the app using an intervention-mapping approach [34] informed by Social Cognitive Theory [35], Self-Determination Theory [36], and the COM-B model [37].

NH staff delivered the sessions and were responsible for creating resident profiles that reflected individual motor and cognitive performance levels and exercise preferences. Based on these profiles, a matching algorithm automatically grouped up to seven residents with comparable abilities and preferences to form small, homogeneous groups for the exercise intervention. The algorithm was based on the motor, cognitive, and exercise preference categories described in the study protocol [33], which also details its validation using simulated resident profiles. All participating NH staff received standardized training covering technical app use, motivational strategies, and adherence procedures. Across facilities, two to five trained staff members delivered the sessions, typically assigned to fixed groups of residents. When substitutions (e.g., due to illness, vacation) were necessary, another trained staff member conducted the session. The app’s step-by-step guidance supported consistent delivery.

Participants engaged in two 30 min group sessions per week over 12 weeks (24 sessions in total). Each session followed a structured format with two to three warm-up exercises emphasizing mobilization and coordination, five to seven main exercises targeting strength, balance, endurance, and gross-motor coordination, and two cool-down exercises focusing on relaxation and body perception. Exercises were selected from approximately 150 app-based activities with adjustable intensity levels and demonstrated through video instructions.

The app supported NH staff through step-by-step on-screen guidance, real-time customization, and post-session feedback, allowing progressive adjustment of exercise difficulty and exclusion of unsuitable activities. Post-session feedback was entered directly in the app, and the system used these ratings to refine subsequent sessions by suggesting more challenging variations for well-tolerated exercises, and omitting exercises rated as unsuitable. Additional features included attendance tracking, a calendar function, integrated health-literacy components, and gamification elements that were rewarded after completion of each session. The gamification elements were intended to support motivation and adherence, consistent with evidence that gamified interventions combined with mHealth technology can increase PA engagement in older adults [38].

### 2.3. Outcomes

Descriptive variables included participants’ age, sex, body mass index (BMI), gait speed, and cognitive status. Cognitive status was assessed using the German version of the Montreal Cognitive Assessment (MoCA) [39,40]. MoCA scores were interpreted using established cut offs: ≥26 indicating no cognitive impairment, 18–25 indicating mild impairment, and ≤17 indicating moderate to severe impairment [39]. Primary outcomes were fall incidence and fall risk. Fall incidence was recorded by NH staff during the 6 months before the intervention, during the 12-week intervention period, and during the 3-month follow-up period postintervention. A fall was defined as “an event in which the individual unintentionally comes to rest on the ground or another lower level” according to the “Expert Standard for Fall Prevention in Nursing Care” of the German Network for Quality Development in Nursing Care [41] (p. 52). Fall data were obtained from routine incident documentation provided by the NH. External verification was not possible, as facilities did not permit access to their internal documentation systems for data protection reasons.

Fall risk was assessed using two performance-oriented balance tests: the Berg Balance Scale (BBS) [42] and the Timed Up and Go test (TUG) [43]. The combined use of the BBS and TUG captures complementary aspects of balance and mobility, which can improve the overall assessment of functional fall risk, as the TUG tends to demonstrate higher sensitivity and the BBS higher specificity [44]. The BBS assesses static and dynamic balance through a 14-item test battery, with each item rated from 0 to 4, with lower scores indicating greater balance impairment (total score range: 0–56). The TUG assesses lower extremity function and mobility, measured as the time in seconds required to rise from a chair, walk three meters, turn, return, and sit down again; walking aids such as canes or rollators were permitted.

The secondary outcome was fear of falling, assessed with the 7-item Short Falls Efficacy Scale-International (Short FES-I) [45], operationalized through a score ranging from 7 to 28, with higher scores indicating more severe concern about falling. All assessments were conducted by trained research assistants who completed a standardized workshop covering all test procedures. To ensure consistency, each assistant administered the same tests across all sites (e.g., two assistants were always responsible for all BBS assessments).

### 2.4. Statistical Analysis

Statistical analyses were conducted in SPSS version 29 (IBM Corp., Armonk, NY, USA). The significance level was set a priori at α = 0.05. Sample size was calculated using G*Power version 3.1.9.7 (Düsseldorf, Germany) [46] based on an effect size of η^2^ = 0.02 derived from results of our group’s literature review of e- and mHealth interventions in nursing homes [27], reflecting the small effects reported therein, power = 0.80, and two groups.

Accounting for a 20% dropout rate and anticipating that not all residents would reach the predefined adherence threshold of 75% for the per-protocol analysis, the initial IG sample was multiplied by 1.5. To achieve equivalent power to an individually randomized RCT, the total sample size was further adjusted by an inflation factor of 1.7 [47], resulting in an a priori calculated sample size of 153 participants in the IG and 102 in the CG. The inflation factor corresponded to an assumed intraclass correlation coefficient (ICC) of 0.05, based on empirical ICC estimates [47] from implementation research datasets.

Despite extended recruitment efforts, the sample size remained slightly below the a priori target due to difficulties with engaging additional NHs within the available study period. The final sample is reported in Section 3.

Baseline differences between the IG and CG were examined using independent-samples *t*-tests, *χ*^2^ tests, or Mann–Whitney U tests, as appropriate. Normally distributed variables are reported as means (*M*) with standard deviations (*SD*). All outcome models were adjusted for sex to account for baseline imbalances. Although BMI also differed between groups, an ANCOVA showed no independent association of BMI with group or sex, while the significant group × sex interaction indicated that the BMI difference reflected the uneven sex distribution rather than a standalone imbalance. Therefore, BMI was not included as a separate covariate.

Changes in fear of falling (Short-FES), balance performance (BBS), and mobility (TUG) were analyzed using linear mixed models (LMMs) with fixed effects for group, time (pre vs. post), and their interaction. Repeated measures (time within residents) were modeled with a compound symmetry covariance structure. For fear of falling, a random intercept for NHs was tested but not retained because the intraclass correlation coefficient was near zero and the model could not produce a stable estimate for this parameter, indicating negligible between-home variability; for BBS and TUG, a random intercept was included. Models were estimated by maximum likelihood with Satterthwaite-adjusted degrees of freedom. Estimated marginal means (EMMs) with Sidak-adjusted pairwise comparisons were reported. Model assumptions for the mixed models were checked and met.

Fall incidence was analyzed separately as follows: Preliminary diagnostics in R [48] (version 4.5.1; packages glmmTMB, DHARMa, performance) using RStudio version 2025.09 (Boston, MA, USA) [49] showed no evidence of zero inflation (*p* = 0.872), supporting a standard negative binomial approach. The main analysis of fall incidence was conducted in IBM SPSS Statistics using Generalized Estimating Equations (GEE) with a negative binomial link. Negative binomial regression analysis is recommended when fitting models for count data that has a Poisson distribution and is over-dispersed [50]. Robust (sandwich) estimators were applied to account for clustering, and exposure time (in days) was included as an offset to account for varying observation periods. Sex was included as a covariate to adjust for the baseline imbalance. Observed incidence rates (IRs) per person-year were calculated from the number of falls and total observation days. Incidence rate ratios (IRRs) with 95% confidence intervals were obtained from the negative binomial GEE model.

Missing data were handled as follows. For continuous outcomes (Short-FES, BBS, TUG), LMMs were estimated using restricted maximum likelihood under the missing-at-random (MAR) assumption, allowing all participants with at least one post-randomization measurement to be retained for the intention-to-treat analysis. Missing data patterns were examined for the two primary outcomes analyzed using LMMs to assess the plausibility of the MAR assumption. Baseline completion rates were 68.5% for TUG and 94.3% for BBS, with non-completion mainly attributable to severe functional limitations (e.g., wheelchair-bound participants unable to stand or transfer independently). Post-assessment completion was 58.1% for TUG and 85.6% for BBS. Among baseline completers, post-assessment missingness was due to documented reasons such as death and hospitalization. Missing TUG and BBS data were associated with poorer baseline functional status, reflected by lower BBS scores and longer TUG times among participants with incomplete data. No systematic differences were observed with respect to age, sex, cognitive status, or group allocation. Overall, missingness was related to observed baseline functional characteristics and documented assessment constraints rather than unobserved outcomes, supporting the MAR assumption.

For fall incidence, missing data were addressed using an available-case approach within the negative binomial GEE framework. Participants without any post-randomization fall information were excluded. Due to the highly skewed distribution of fall counts and the presence of zeros, multiple imputation was not performed.

All main analyses were repeated as per-protocol analyses, restricted to IG participants with ≥75% adherence and all controls.

## 3. Results

### 3.1. Descriptive Statistics

**Figure 1:**
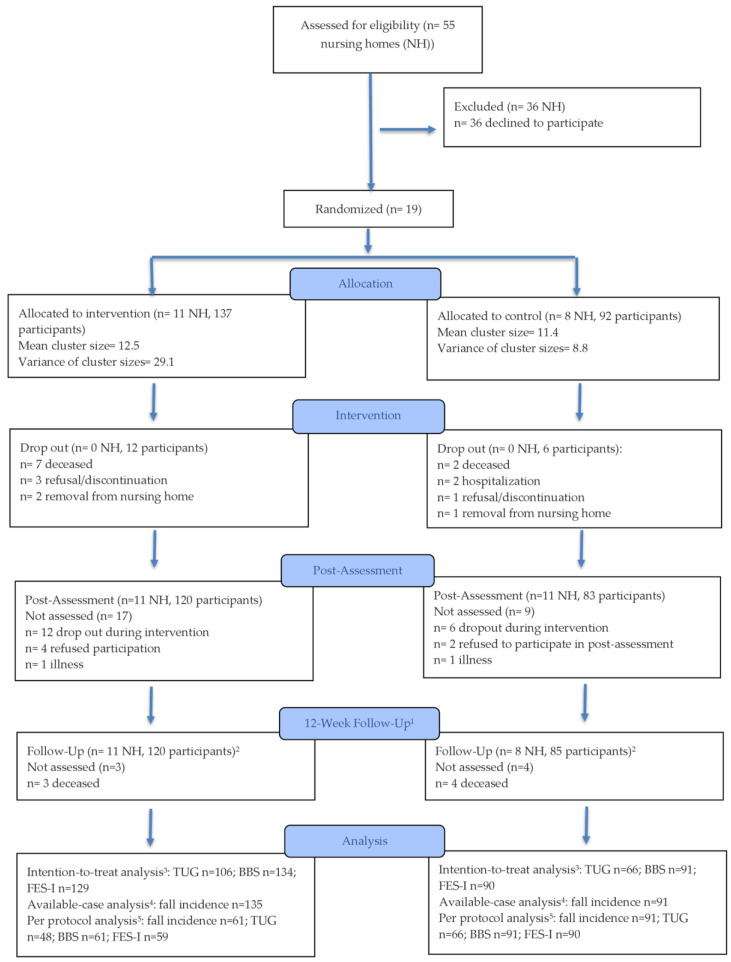
CONSORT Flow Diagram. ^1^ Only number of falls assessed. ^2^ Includes participants who could not attend post-assessment (e.g., due to illness, hospitalization). ^3^ Includes all randomized participants with at least one post-randomization observation (LMM using available data under MAR assumption). ^4^ Includes all randomized participants with at least one post-randomization fall record (GEE using available cases only). ^5^ Includes all randomized participants with at least one post-randomization observation (LMM using available data under MAR assumption) with ≥75% session adherence in the intervention group and all control participants with at least one post-randomization observation.

A total of 55 NHs were assessed for eligibility between October 2023 and May 2024. Nineteen facilities were randomized to the IG or CG, resulting in 229 participating residents (171 females; 74.7%). Of these, 137 residents were allocated to the IG and 92 to the CG. The participant flow is illustrated in Figure 1.

The mean age of residents was 85.4 years (*SD* = 7.4; range 65–99 years), and the mean BMI was 27.2 kg/m^2^ (*SD* = 5.6). On average, residents showed moderate cognitive impairment (MoCA score: M = 14.4, *SD* = 6.8) and used 8.9 medications (*SD* = 4.1). Statistically significant baseline differences between IG and CG were found for sex distribution (*p* < 0.01) and BMI (*p* = 0.02). Detailed sample characteristics are provided in Table 1, and a more comprehensive description of the study sample is available in [33].

### 3.2. Adherence to the Program

On average, employees delivered 85.6% of the intended sessions (20.5 out of the 24). Residents participated in 75.1% of the sessions provided (mean: 18.0 sessions; adjusted adherence), and 64.3% of the originally planned sessions (mean: 15.4 sessions; unadjusted adherence). Further details regarding program acceptance by NH staff and residents are reported elsewhere [51].

### 3.3. Effectiveness of the Program

**Figure 2:**
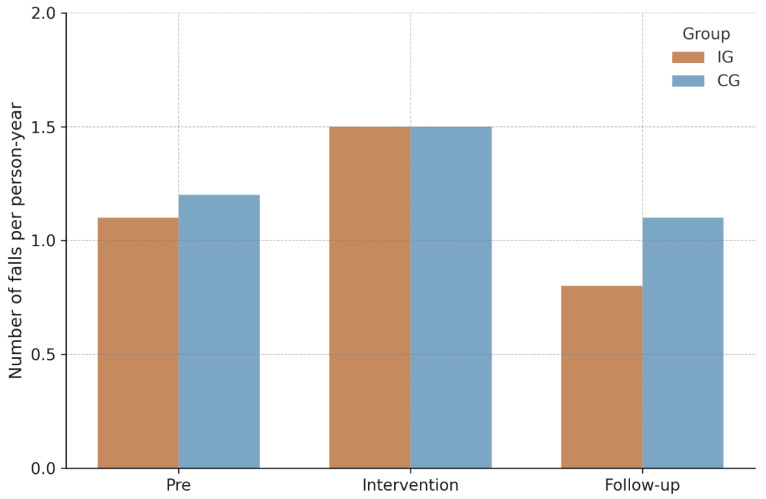
Fall rate per person-year. Note. IG = Intervention group; CG = Control group.

For fall incidence, negative binomial GEE models (see Table 2) showed no statistically significant group × time interaction, Wald *χ*^2^(2) = 0.67, *p* = 0.72, indicating no differential change between IG and CG over time. A small main effect of time emerged, Wald *χ*^2^(2) = 6.07, *p* = 0.048, whereas the main effect of group was nonsignificant, Wald *χ*^2^(1) = 0.92, *p* = 0.34. Observed IRs per person-year are visualized in Figure 2, and corresponding model-based IRRs are summarized together with IRs in Table 3. During the intervention phase, IRs were 1.5 (95% CI [1.0–2.0]) in the IG and 1.5 (95% CI [1.0–2.2]) in CG (IRR = 0.9, 95% CI [0.5–1.6], *p* = 0.83). In the follow-up phase, rates measured 0.8 (95% CI [0.5–1.2]) and 1.1 (95% CI [0.7–1.6]) respectively (IRR = 0.5, 95% CI [0.2–1.2], *p* = 0.14). Similar non-significant findings were observed in the per-protocol analysis restricted to participants with ≥75% adherence.

The results of the LMM analyses are presented in Table 4. Analysis of the TUG test showed no statistically significant group × time interaction, *F*(1, 104.45) = 0.01, *p* = 0.91, 95% CI [−4.15, 4.66], indicating no differential change in TUG times between groups. Neither the main effect of group, *F*(1, 13.61) = 0.92, *p* = 0.36, nor time, *F*(1, 104.45) = 0.99, *p* = 0.32, reached significance. EMMs showed similar performance over time in both groups (intervention: 28.8 s to 29.8 s; control: 35.1 s to 36.3 s).

Analysis of the BBS showed a statistically significant group × time interaction, F(1, 190.81) = 8.25, *p* = 0.005, 95% CI [−5.98, −1.11], indicating differential change between groups. Neither the main effect of group, *F*(1, 14.41) = 0.01, *p* = 0.934, nor time, *F*(1, 190.81) = 0.28, *p* = 0.595, reached significance. EMMs showed that the IG improved from 27.1 to 28.5 points, whereas the control group declined from 28.6 to 26.5 points. The magnitude of this differential change corresponds to a standardized mean difference of *d* = 0.22, indicating a small effect.

The Short FES-I showed no statistically significant group × time interaction, *F*(1, 189.24) = 1.93, *p* = 0.17, 95% CI [−0.37, 2.13], indicating no differential change in fear of falling between groups. Main effects of time and group were also nonsignificant. EMMs indicated similar scores across time in both groups (intervention: 11.6 to 11.2 points; control: 11.4 to 11.9 points). At baseline, 20.5% of participants scored at the minimum Short FES-I value of 7, reflecting the best possible score and indicating a ceiling effect with limited scope for detectable improvement.

Results from the per-protocol analyses were consistent with the main findings. The group × time interaction remained nonsignificant for TUG performance, *F*(1, 76.69) = 0.05, *p* = 0.82, 95% CI [−5.99, 4.78] and Short FES-I, *F*(1, 131.56) = 0.90, *p* = 0.37, 95% CI [−0.77, 2.06]. In contrast, the interaction for the BBS remained statistically significant, *F*(1, 138.23) = 10.62, *p* = 0.001, 95% CI [−7.85, −1.92], *d* = 0.30. Please refer to Table 5 for the results of the per protocol analysis.

## 4. Discussion

This cRCT evaluated the effectiveness of a multicomponent exercise program delivered using the tablet-based BeSt Age App for reducing falls and fall risk among NH residents. While the intervention significantly improved balance performance as measured by the BBS, no effects were observed on mobility (TUG), fear of falling (Short FES-I), or fall incidence. These findings partially support the hypothesis that a staff-delivered mHealth exercise program can improve functional components of fall risk, though its impact on actual fall rates appears limited within the study timeframe.

The improvement in BBS performance suggests that the intervention effectively enhanced residents’ static and dynamic balance, which is a key component of fall risk [52,53]. This finding aligns with evidence that multicomponent exercise programs combining balance, strength, and mobility training can improve postural control [54]. Although no established minimal clinically important difference for the BBS exists for NH populations, reported values in clinical groups range from approximately 2 to 12 points depending on diagnosis and functional level [55,56,57,58,59]. While these estimates are not directly transferable to NH residents, the observed 3.5-point between-group difference falls within the lower bound of these ranges, suggesting potential clinical relevance. The absence of a corresponding improvement in TUG performance may reflect the distinct physical and cognitive demands of the test, which assesses gait and transfer speed. In addition, TUG scores can vary substantially depending on the use and type of walking aid, introducing measurement variability that may have obscured potential intervention effects [43,60].

Overall, these results indicate that the intervention improved specific elements of balance control but not the broader functional mobility domain. Although the BBS and TUG are established indicators of functional fall risk, their predictive validity for actual fall events is limited, and cut-off values vary considerably across populations [60,61,62]. Consequently, the observed BBS improvement should be interpreted as a functional gain in balance within the broader multifactorial construct of fall risk.

Despite improved balance, no reduction in fall incidence was observed. This dissociation between improved test performance and actual fall reduction is consistent with previous research showing that enhanced physical function does not necessarily translate into fewer falls [63,64]. Falls in NHs are shaped by numerous non-physical determinants such as medication use and environmental hazards [65]. Together with physical and cognitive determinants, this multicausal interplay helps explain why improvements in isolated functional domains may not translate directly into reductions in fall incidence. Resident characteristics, including frailty and cognitive impairment, appear to moderate intervention effects and may attenuate or even counteract expected benefits [64]. Exercise frequency (≥3 sessions per week vs. <3 sessions per week) may not substantially influence intervention effectiveness, although this evidence is of low certainty [64]. Beyond frequency, total intervention duration seems to be a decisive factor for fall prevention effectiveness. The present 12-week program likely provided sufficient stimulus to elicit short-term balance improvements, but not the prolonged exposure required to affect fall incidence. Previous studies indicate that interventions lasting six months or longer are most effective in reducing falls among NH residents [16,64]. In addition to intervention frequency and duration, the nature of the training stimulus may also play a critical role. Conventional balance exercises typically emphasize controlled, predictable movements, which may not fully prepare individuals for unexpected balance perturbations encountered in daily life. Incorporating more task-specific and ecologically valid balance exercises, such as perturbation-based or reactive balance training, could therefore help bridge the gap between functional improvements and real-world fall prevention [66].

The absence of an intervention effect on fear of falling in this study is consistent with previous findings from long-term care settings, where exercise-based interventions alone have shown limited effects. Lach and Parsons reported that most studies investigating fear of falling in NH residents used balance and strength training but rarely achieved significant improvements, likely due to small samples and low baseline fear of falling [67]. In our study, baseline fear of falling scores were also low, with many residents scoring at the minimum value, indicating a floor effect that might have constrained detectable change. At the same time, evidence from previous research indicates that programs combining exercise with cognitive-behavioral strategies can reduce fear of falling and fall incidence, highlighting the importance of psychological components [68]. Evidence from community-dwelling older adults supports this pattern: meta-analyses show that multicomponent programs including both exercise and cognitive-behavioral elements are more effective than exercise alone [69,70]. Overall, while exercise can improve physical determinants of fall risk, psychological factors appear to play an important role, suggesting that meaningful reductions in fear of falling may require integrative behavioral approaches.

These findings suggest that technology-assisted, staff-mediated programs may offer a viable approach to promoting sustainable PA in institutional care. The present study demonstrated that such an mHealth-based intervention is feasible and can enhance balance performance among NH residents. By supporting NH staff in the preparation and delivery of exercise sessions, the BeSt Age App facilitated implementation and reduced organizational barriers [51]. Unlike most previous digital interventions that excluded frail residents [27], this study showed that a staff-guided mHealth approach can be successfully implemented even among individuals with significant physical or cognitive limitations. However, the inclusion of NH residents with higher frailty levels may have limited the intervention’s effectiveness on mobility and fall incidence. However, the inclusion of more frail residents may have limited effects on mobility and fall incidence, as frailty is linked to impaired vascular responsiveness [71], higher risk of adverse advents that can hinder adherence [72], declines in gait plasticity that might constrain mobility gains [73] and a greater likelihood of physical overload during exercise [64].

Key strengths of this study include its cRCT design and use of validated outcome measures. Robust statistical methods accounted for clustering and exposure time, ensuring appropriate inference. Moreover, by including NH residents with advanced physical and cognitive impairments, this study captures a more inclusive and representative NH population, thereby enhancing external validity and practical relevance. Several limitations should be acknowledged. The relatively short intervention and follow-up durations may have limited the ability to detect changes in fall incidence. The final sample size was slightly lower than planned due to recruitment challenges, which may have marginally reduced statistical power. Fall reporting relied on NH staff documentation with no possibility for independent confirmation by the research team, which means that variation in documentation practices across sites and shift teams could have resulted in incomplete or inconsistent reporting. Finally, while TUG and BBS capture important components of fall risk, they may not fully represent the multifactorial etiology of falls in institutionalized populations.

## 5. Conclusions

In summary, the BeSt Age App improved balance performance in a 12-week cRCT, but did not have a statistically significant impact on fall incidence, mobility, or fear of falling. These findings highlight that NH staff-delivered multicomponent mHealth exercise programs may improve physical capacity in NH residents, even in the presence of high frailty levels. Future studies should evaluate longer intervention durations, integration with behavioral and environmental fall prevention components, and the inclusion of task-specific, ecologically valid balance exercises that better translate functional gains into real-world fall prevention. Building on the implementation-science elements already integrated into the BeSt Age project, future research could further expand such approaches to systematically address contextual factors and organizational readiness, which are critical for sustainable delivery in long-term care settings.

## Figures and Tables

**Table 1:** Participant characteristics (nursing home residents) at baseline.

Variable	Intervention Group (*n* = 137)	Participants with Missing Information	Control Group (*n* = 92)	Participants with Missing Information	Total (*N* = 229)	Participants with Missing Information	*p*
Female sex, n (%)	111 (81)	—	60 (65.2)	—	171 (74.7)	—	<0.01
Age (years)	85 (7.6)	2	85.9 (7.2)	—	85.4 (7.4)	2	0.31
BMI (kg/m^2^)	28.1 (5.8)	8	25.9 (5)	5	27.2 (5.6)	13	0.02
MoCA (points)	13.9 (6.2)	12	15.1 (5.7)	1	14.4 (6.8)	13	0.18
BBS (points)	26.5 (16.3)	11	28.9 (15.9)	2	27.2 (16.2)	13	0.41
TUG (s	27.6 (11.7)	44	28.3 (16.5)	29	27.9 (13.8)	73	0.53
Short FES-I (points)	11.6 (4.5)	12	11.4 (4.2)	1	11.5 (4.4)		0.75
10MWT (m/s)	0.6 (0.3)	25	0.6 (0.3)	10	0.6 (0.3)	35	0.37
Number of falls (6 months before baseline)	0.5 (1.6)	16	0.6 (1.6)	2	0.6 (1.5)	18	0.85
Number of drugs	8.7 (3.9)	5	9.1 (4.5)	20	8.9 (4.1)	25	0.57

Note. Values are presented as mean (*SD*) unless otherwise indicated. BMI: Body Mass Index; MoCA: Montreal Cognitive Assessment; BBS: Berg Balance Scale; TUG: Timed Up and Go Test; Short FES-I: Short Falls Efficacy Scale–International; 10MWT: 10 m walking test (m/s) at preferred pace.

**Table 2:** Generalized Estimating Equation Results (Negative Binomial Link) for Fall Incidence: Available-Case (IG *n* = 135, CG *n* = 91) and Per-Protocol Analyses (IG *n* = 61, CG *n* = 91).

Effect	*χ* ^2^	*df*	*p*
Available-case analysis			
Group × Time	0.67	2	0.72
Time	6.07	2	0.048
Group	0.92	1	0.34
Per-protocol analysis			
Group × Time	0.49	2	0.78
Time	4.85	2	0.089
Group	0.58	1	0.45

Note. GEE models with negative binomial distribution and log link were fitted with observation days as the exposure term and adjusted for sex. Robust (sandwich) estimators were applied to account for clustering.

**Table 3:** Observed incidence rates (with 95% CI) and model-based incidence rate ratios (IRRs) per person-year.

Phase	Group	*n*	Total Falls (Observation Days)	IR (95% CI)	IRR * (95% CI)	*p*
Pre	IG	131	71 (182.5)	1.1 (0.8–1.4)	0.8 (0.5–1.4)	0.621
	CG	90	54 (182.5)	1.2 (0.9–1.6)	1 (reference)	
Intervention	IG	106	36 (84.0)	1.5 (1.0–2.0)	0.9 (0.5–1.6)	0.830
	CG	81	28 (84.0)	1.5 (1.0–2.2)	1 (reference)	
Follow-up	IG	120	24 (91.25)	0.8 (0.5–1.2)	0.5 (0.2–1.2)	0.140
	CG	85	23 (91.25)	1.1 (0.7–1.6)	1 (reference)	

Note. IR = incidence rate per person-year; IRR = incidence rate ratio (Intervention vs. Control). * IRRs are model-based (negative binomial General Estimation Equations (GEE)), adjusted for sex.

**Table 4:** Results of the Linear Mixed Model Analyses (Intention-to-Treat) TUG.

Outcome	Group (*n*)	Pre-Test *M* (*SE*)	Post-Test *M* (*SE*)	*F*(1, *df*_2_)	*p*	95% CI (ΔIG–−CG)
TUG (s)	IG (106)	28.8 (4.2)	29.8 (4.2)	0.01 (1, 104.45)	0.91	[−4.15, 4.66]
	CG (66)	35.1 (5.3)	36.3 (5.3)			
BBS (points)	IG (134)	27.1 (1.9)	28.5 (1.9)	8.25 (1, 190.81)	0.005	[−5.98, −1.11]
	CG (91)	28.6 (2.3)	26.5 (2.3)			
Short-FES-I (points)	IG (129)	11.6 (0.4)	11.2 (0.4)	1.93 (1, 189.24)	0.17	[−0.37, 2.13]
	CG (90)	11.4 (0.5)	11.9 (0.5)			

Note. TUG = Timed Up and Go Test; BBS = Berg Balance Scale; Short-FES-I = Short Falls Efficacy Scale-International; IG = intervention group; CG = control group. Values are estimated marginal means (*M*) with standard errors (*SE*) in parentheses. Reported statistics refer to the group × time interaction.

**Table 5:** Results of the Linear Mixed Model Analyses (Per-Protocol).

Outcome	Group (*n*)	Pre-Test *M* (*SE*)	Post-Test *M* (*SE*)	*F*(1, *df*_2_)	*p*	95% CI (ΔIG–−CG)
TUG (s)	IG (48)	29.1 (7.4)	30.8 (7.4)	0.05 (1, 76.69)	0.82	[−5.99, 4.78]
	CG (66)	34.8 (6.3)	35.9 (6.4)			
BBS (points)	IG (61)	27.9 (2.9)	30.7 (2.9)	10.62 (1, 138.23)	0.001	[−7.85, −1.92]
	CG (91)	28.6 (2.5)	26.5 (2.5)			
Short-FES-I (points)	IG (59)	11.3 (0.5)	11.1 (0.6)	0.90 (1, 131.56)	0.37	[−0.77, 2.06]
	CG (90)	11.4 (0.4)	11.9 (0.5)			

Note. TUG = Timed Up and Go Test; BBS = Berg Balance Scale; Short-FES-I = Short Falls Efficacy Scale-International; IG = intervention group; CG = control group. Per-protocol analysis includes only intervention participants with ≥75% adherence; all controls retained. Values are estimated marginal means (*M*) with standard errors (*SE*) in parentheses. Reported statistics refer to the group × time interaction effects.

## Data Availability

The original contributions presented in this study are included in the article. Further inquiries can be directed to the corresponding author.

## Abbreviations

The following abbreviations are used in this manuscript:

BBS	Berg Balance Scale
BMI	Body Mass Index
cRCT	Cluster-Randomized Controlled Trial
CG	Control Group
EMMs	Estimated Marginal Means
GEE	Generalized Estimating Equations
IG	Intervention Group
IRs	Incidence Rates
IRRs	Incidence Rate Ratios
LMMs	Linear Mixed Models
MAR	Missing at Random
MoCA	Montreal Cognitive Assessment
NH	Nursing home
PA	Physical Activity
Short FES-I	Short Falls Efficacy Scale-International
TUG	Timed Up and Go Test
